# Comprehensive Library Generation for Identification and Quantification of Endometrial Cancer Protein Biomarkers in Cervico-Vaginal Fluid

**DOI:** 10.3390/cancers13153804

**Published:** 2021-07-28

**Authors:** Kelechi Njoku, Davide Chiasserini, Bethany Geary, Andrew Pierce, Eleanor R. Jones, Anthony D. Whetton, Emma J. Crosbie

**Affiliations:** 1 Division of Cancer Sciences, Faculty of Biology, Medicine and Health, University of Manchester, 5th Floor Research, St Mary’s Hospital, Oxford Road, Manchester M13 9WL, UK; kelechi.njoku@manchester.ac.uk (K.N.); eleanor.jones-3@manchester.ac.uk (E.R.J.); 2 Department of Obstetrics and Gynaecology, Manchester University NHS Foundation Trust, Manchester Academic Health Science Centre, Manchester M13 9WL, UK; 3 Stoller Biomarker Discovery Centre, Division of Cancer Sciences, Faculty of Biology, Medicine and Health, University of Manchester, Manchester M13 9PL, UK; davide.chiasserini@unipg.it (D.C.); bethany.geary@manchester.ac.uk (B.G.); 4 Wolfson Molecular Imaging Centre, Division of Cancer Sciences, Faculty of Biology, Medicine and Health, University of Manchester, Palatine Road, Manchester M20 3LJ, UK; andrew.pierce@manchester.ac.uk; 5 Department of Medicine and Surgery, Section of Physiology and Biochemistry, University of Perugia, 06132 Perugia, Italy

**Keywords:** endometrial cancer, spectral library, cervico-vaginal fluid, non-invasive, proteomics, protein, peptide, mass spectrometry, SWATH-MS

## Abstract

**Simple Summary:**

Endometrial cancer is the most common cancer of the female reproductive tract, and its incidence is rising. Early diagnosis has the potential to improve survival as women can receive care at the earliest possible stage when curative treatment is likely. Current tests for endometrial cancer diagnosis are sequentially invasive with low patient acceptability. A detection tool based on minimally invasive samples such as cervico-vaginal fluid would be a major advance in the field. This study focuses on the potential of detecting endometrial cancer based on the proteins and peptides expressed in cervico-vaginal fluid. Using Sequential window acquisition of all theoretical mass spectra (SWATH-MS), we present a spectral library of thousands of proteins in the cervico-vaginal fluid of women with or at risk of endometrial cancer. This important resource will enable the identification of endometrial cancer biomarkers in cervico-vaginal fluid and advances our knowledge of the role of proteomics in endometrial cancer detection.

**Abstract:**

Endometrial cancer is the most common gynaecological malignancy in high-income countries and its incidence is rising. Early detection, aided by highly sensitive and specific biomarkers, has the potential to improve outcomes as treatment can be provided when it is most likely to effect a cure. Sequential window acquisition of all theoretical mass spectra (SWATH-MS), an accurate and reproducible platform for analysing biological samples, offers a technological advance for biomarker discovery due to its reproducibility, sensitivity and potential for data re-interrogation. SWATH-MS requires a spectral library in order to identify and quantify peptides from multiplexed mass spectrometry data. Here we present a bespoke spectral library of 154,206 transitions identifying 19,394 peptides and 2425 proteins in the cervico-vaginal fluid of postmenopausal women with, or at risk of, endometrial cancer. We have combined these data with a library of over 6000 proteins generated based on mass spectrometric analysis of two endometrial cancer cell lines. This unique resource enables the study of protein biomarkers for endometrial cancer detection in cervico-vaginal fluid. Data are available via ProteomeXchange with unique identifier PXD025925.

## 1. Introduction

Endometrial cancer (EC) is the sixth most common malignancy and the 14th leading cause of cancer-related mortality in women globally [1]. Over 380,000 incident cases were diagnosed in 2018 and approximately 89,000 succumbed to their disease. Its incidence is projected to escalate in the coming years in tandem with the rising prevalence of obesity [2,3]. Over 80% of endometrial cancers are what Bokhman termed type 1 EC, where low grade cancers, often preceded by a precursor lesion (atypical hyperplasia (AH)), develop from a background of unopposed oestrogen. Type 1 tumours generally have a favourable prognosis compared with the less common, high grade, oestrogen-independent and clinically aggressive type 2 EC [4,5]. A more pragmatic classification of EC by the Cancer Genome Atlas Research Network categorises EC into four distinct molecular subtypes: polymerase epsilon (*POLE*) ultramutated, microsatellite instable (MSI), copy number low and copy number high, and this has been shown to have prognostic implications in multiple studies [6,7].

While over 90% of women with EC initially present with postmenopausal bleeding (PMB), only 5–10% of women with PMB have EC [8,9]. Early detection of EC is crucial for good survival outcomes and will enable conservative treatment options to be offered, for example, to women in whom surgery is potentially hazardous [10,11]. Current EC diagnostic modalities such as transvaginal ultrasound scan (TVS) and endometrial biopsy have limitations in terms of sub-optimal specificity (TVS) and reduced acceptability from invasiveness and discomfort (biopsy) [11,12]. Given the anatomical continuity of the uterine cavity with the lower genital tract, there is growing interest in the potential for minimally-invasive sampling methodologies to enable EC detection [13]. Proximal fluids, such as endometrial secretions, derive from or are in direct contact with the diseased organ and are therefore a reliable source of cancer-derived biomarkers [14]. The dynamic range of biomarkers in proximal fluids is significantly reduced as compared to plasma, allowing better sensitivity for the detection of biologically relevant molecules [15,16].

In recent years, there has been growing interest in the use of high-throughput technologies for cancer biomarker discovery [14,17]. Platforms such as mass spectrometry (MS) based proteomics, for instance, have shown promise for facilitating the comprehensive analysis of complex biological specimens to better understand both physiological and pathological states [18]. Although transcriptomic analyses can also be used to infer cellular activities, they do not truly reflect the functional phenotype of the cell nor directly correlate to the proteome. Proteomic analysis, on the other hand, details the phenotype or endotype directly [15]. 

An emerging MS-based technology of high precision and accuracy that is used to identify and quantify proteins is the sequential window acquisition of all theoretical mass-spectra (SWATH-MS). SWATH-MS is highly reproducible and is a data-independent acquisition (DIA) methodology with a peptide-centric data analytical strategy [19]. This proteomic technique requires a spectral library of peptide transitions in order to identify and quantify peptides from the multiplexed mass spectrometry data. The robustness of the spectral library significantly impacts on the study outcome as a peptide needs to be present in an assay library for it to be identified and quantified by SWATH MS [20]. Developing a high quality library that contains all the peptide components that are likely to be extracted from the MS data is therefore critical to the success of any SWATH MS-based study [19].

Here, we present a bespoke spectral library for SWATH-MS based quantification of proteins expressed in the cervico-vaginal fluid of postmenopausal women with, or at risk of EC. Using a data-dependent acquisition MS analysis, we generated data on 18,247 proteotypic peptides. The assay also includes data on precursor and fragment ion *m/z*, relative ion intensities and normalised retention times. Cervico-vaginal samples were obtained using a Delphi screener, a sterile, pre-packaged plastic device that is specifically tailored for cervico-vaginal sample collection and works by a lavage principle. A library based on two EC cell-lines was also generated and consists of 27,972 proteotypic peptides and 6003 proteins. This approach will enable biomarker discovery and validation to inform development of an innovative point of care test via a unique catalogue of peptides and proteins in the cervico-vaginal fluid of women with or at risk of EC. In addition, by making the raw MS data available, we offer the opportunity for data re-use and analysis with other state-of-the-art analytical software. 

## 2. Methods

### 2.1. Research Ethics and Approval

**Figure 1:**
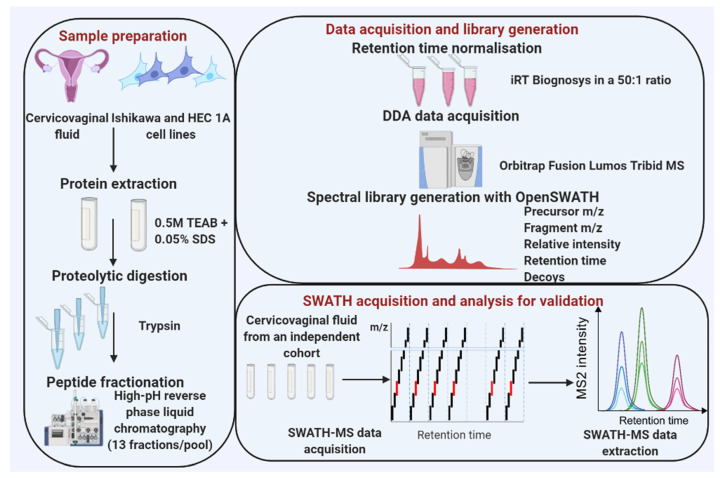
An overview of data acquisition and spectral library generation workflow. Data acquisition: Pooled cervico-vaginal fluid samples from 9 cancer cases, 3 AH and 7 controls were used for the library generation while two EC cell lines were used for the cell line-based library. Cervico-vaginal samples were separated into pellets and supernatants. Pellets from both cervico-vaginal fluid and EC cell lines were subjected to lysis and protein extraction. All samples were subjected to proteolytic digestion with trypsin, peptide fractionation using high-pH reverse phase liquid chromatography and LC-MS/MS analysis. Library generation: Sequence database search was done using X!Tandem while the library construction was performed using the OpenSWATH pipeline. Library validation: technical replicates of five cervico-vaginal samples were used to search the library to assess its applicability for SWATH-MS data analysis, while ten samples (five cancers and five controls) were used to assess the potential utility of the library for biomarker discovery in the real-world setting.

**Figure 2:**
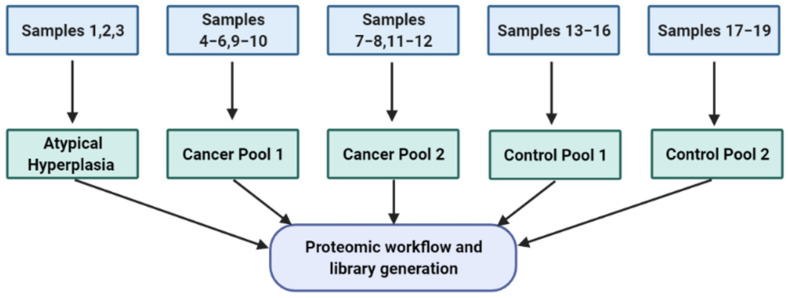
Sample pool strategy used for library generation. This is based on both cervico-vaginal supernatant and pellet samples. Three AH samples were pooled together while two pools were made from five cancer samples (cancer pool 1) and 4 cancer samples (cancer pool 2) respectively. The seven control samples were also pooled in two, based on four samples (control pool 1) and three samples (control pool 2) respectively. List of pooled samples used in library generation are summarised in Table 2.

Cervico-vaginal samples from women with AH/EC (cases) and from a similar at-risk population who do not have EC (controls) were pooled and used for the library generation. Eligibility for inclusion is as summarised in Table 1. This study was approved by the North-West Greater Manchester Research Ethics Committee (reference-16/NW/0660). All subjects provided written informed consent prior to the collection of biological samples. Samples were collected and stored anonymously in accordance with the principles of the Declaration of Helsinki and in compliance with the Human Tissue Act, 2004. All data generated from participants medical history and collected samples were handled and stored in accordance with the data protection principles of the General Data Protection Regulation (GDPR) 2018. Two EC cell lines (Ishikawa and HEC-1A) were used for the cell-lined based assay library generation. The experimental workflow is shown in Figure 1 and the sample pool scheme in Figure 2.

### 2.2. Sample Collection

Cervico-vaginal samples were collected using a Delphi screener, a plastic device which is approximately 20 cm long. With participants lying supine, legs apart, knees bent and heels brought up to the bottom, the device was inserted into the vagina and the plunger pressed, releasing a small reservoir of sterile saline fluid into the vagina. Pressure was then taken off the plunger and the fluid collected back into the device, which was then removed. Following collection, samples were centrifuged at 1000× *g* for 10 min to separate cellular pellets from supernatant fractions, which were stored separately at −80 degrees. The pellets were treated with 1 mL of red blood cell (RBC) lysis solution (BD CytoRich Red, Becton Dickinson, NJ, USA, re-suspended by gentle pipetting, incubated for 5 min at room temperature and centrifuged at 1000× *g* for 10 min. The RBC lysis supernatant was then discarded, and the pellets washed by centrifugation at 1000× *g* for 5 min with phosphate buffered saline prior to storage at −80 °C. 

### 2.3. Cell Culture

EC cell lines (Ishikawa and HEC1A) were obtained, authenticated and tested for Mycoplasma prior to usage. Cell lines were cultured in DMEM growth medium (Gibco Life Technologies; Thermo Fisher Scientific, Inc., Waltham, MA, USA) supplemented with 10% (*v*/*v*) heat inactivated fetal bovine serum (Hi-FBS) (Gibco Life Technologies) and 2 mM glutamine (GlutaMAX). Cells were grown in 25 cm^2^ or 75 cm^2^ culture flasks (Corning, Tewksbury, MA, USA) and maintained at 37 °C, 95% humidity and 5% (*v*/*v*) CO_2_ (Thermo Fisher, Waltham, MA, USA). At 80% confluence, both cell lines were harvested, and a cell count conducted using a haemocytometer. Subculture was conducted multiple times and harvested cells pooled and pelleted by centrifugation at 1500× *g* for five minutes and stored at −80 degrees pending further analyses. 

### 2.4. Cervico-Vaginal Fluid Supernatant Preparation

The cervico-vaginal supernatants were concentrated using the Agilent spin concentrator (4 mil 30K MWCO concentrator, Agilent UK, Cheadle, UK). Using the same spin, buffer exchange with 25 mM ammonium bicarbonate was performed prior to protein assay.

### 2.5. Cell Lysis/Protein Extraction

Pellets from both EC cell lines and cervico-vaginal samples were lysed in 0.5 M TEAB buffer with 0.05% (*w*/*v*) SDS (Protease and Phosphatase inhibitor cocktail and Benzonase) and incubated for 30 min at 4 degrees. Lysates were vortexed briefly every 10 min and sample checked until clear of DNA. Cell line pellets were lysed in 50 uL of lysis buffer per 10^6^ cells while cervico-vaginal sample pellets were lysed in 500 uL of buffer. Samples were centrifuged at 10,000× *g* at 4 degrees for 10 min and supernatants collected in pre-chilled Eppendorf vials. 

### 2.6. Protein Digestion

Protein concentration was measured using the Bradford assay (Bio-rad laboratories, Watford, UK). Appropriate volumes of cervico-vaginal fluid and EC cell lines lysates containing 20 µg (x19) and 30 µg of protein respectively were transferred into clean Eppendorf vials. Disulphide bonds were reduced by the addition of 0.1 volumes of 50 mM tris-(2-carboxyethyl) phosphine (TCEP) to the fluid and incubation in a heating block at 60 degrees for 1 h. Alkylation was performed using 10 mM iodoacetamide in the dark at room temperature for 30 min and digestion completed with trypsin (Promega, Southampton, UK) at a 10:1 protein: trypsin ratio and incubated overnight at 37 °C.

### 2.7. High-pH Fractionation of Peptides

Peptide fractionation was performed using a high pH reverse phase liquid chromatography system (Agilent). Sample peptides were resuspended in 1 mL of a mixture of 97% Buffer A (0.1% (*v*/*v*) ammonium hydroxide in water adjusted to pH 10.5) and 3% Buffer B (0.1% (*v*/*v*) ammonium hydroxide in pure acetonitrile adjusted to pH 10.5) and loaded onto a C18 reverse phase (RP) column. Peptides were eluted at 700 µL/min with a 30-min linear gradient of acetonitrile (Fisher scientific) starting from 0.5% (*v*/*v*) buffer B to 50% (*v*/*v*). A total of 96 fractions were collected (30 s each) for a duration of ~30 min per run. The collected samples were concatenated in 12 fractions + the flow through. Peptides were subsequently dried using a speed-vac (Genevac) and stored at −80 °C. 

### 2.8. DDA Mass Spectrometry for Spectral Library Generation

For library construction, dried peptides were vigorously resuspended with 40 μL of loading buffer (2%ACN/0.1% formic acid and 10x iRT Biognosys in a 50:1 ratio). 5 μL (~1 μg) of each peptide sample were subsequently trapped in a 10 cm fused silica Acclaim™ PepMap™ 5 μm 100 Nano-Trap Column (Thermo Scientific, Waltham, MA, USA) and equilibrated with 2% buffer B (0.1% FA in 80% ACN) for 15 min at a flow rate of 300 nL/min prior to increasing the concentration of Buffer B to 7% over 5 min and a 90 min. gradient (2–45% buffer B at 300 nL/min) using a U3000 RSLC high pressure nanoLC (Dionex). The column was re-equilibrated with 2% buffer B before the next injection. Eluting peptides were measured on-line by an Orbitrap Fusion Lumos Tribrid mass spectrometer (Thermo Scientific), operating in data-dependent acquisition mode. Peptides were ionized using a coated silica emitter at a potential of +1.9 kV (Thermo Fischer Scientific) and focused using an S-lens value of 60. Intact peptide ions were detected at a resolution of 60,000 (at *m/z* 200) and fragment ions at a resolution of 30,000 (at *m/z* 200); the MS mass range was 350–1500 Da. Automatic gain control target settings for MS were 4 × 10^5^ charges and for MS/MS 1 × 10^5^ charges, with a maximum injection time of 50 and 120-ms respectively. Peptides were selected for higher-energy C-trap dissociation fragmentation at an underfill ratio of 1% and a quadrupole isolation window of 1.5 Da. Precursor ions were fragmented at a normalized collision energy of 28.

### 2.9. Building the SWATH Spectral Library

All LC-MS/MS data obtained from the Orbitrap Fusion Lumos Tribrid mass spectrometer (Data Citation 1) were converted into an mzXML format, centroided and searched using X!Tandem (version PILEDRIVER-2015/04/01) against the full 20,271 non-redundant canonical sequences (without isoforms) of the UniProtKB/Swiss-Prot database (17 December 2015) appended with iRT peptides and decoy sequences. The datasets (cervico-vaginal and cell lines) were searched individually using the default X!Tandem search parameters with few modifications as follows: a maximum valid expectation value of 10; fixed residue mass modification of +57.022 for carbamidomethylated cysteine; variable residue mass modification of +16.0 for oxidized methionine; enzyme specificity = trypsin; spectrum parameters including a fragment monoisotopic mass error of 40 ppm and a parent mono-isotopic mass error of ±15 ppm; spectrum conditioning parameters of 100.0 spectrum dynamic range, total spectrum peaks 50, a minimum parent (M + H) + of 400.0, and a minimum fragment *m/z* of 150. SpectraST, operating in a library generation mode, was used for consensus library generation while the TargetedFileConverter tool from the OpenSWATH workflow was used to convert TSV files to TraML format. Decoys were subsequently appended to the TraML assay using the OpenSwathDecoyGenerator tool operating in a reverse mode with an identity threshold of 1 and a similarity threshold of 0.05 Da. The consensus assay library was subsequently converted into a Transitions Markup Language (TraML) file format for downstream analysis. 

### 2.10. Assay Library False Discovery Rate Control

We applied a target decoy database to adjust the assay library to an FDR of 1%, giving rise to a 0.99% probability cut-off [21].

### 2.11. SWATH-MS Acquisition and Library Validation

MS analysis of cervico-vaginal fluid samples used for library validation was performed using a 6600 TripleTOF (Sciex, Warrington, UK). The LC method was based on a 120-min gradient between a buffer A of 98% Water, 2% (*v*/*v*) Acetonitrile and 0.1% (*w*/*v*) Formic Acid and a buffer B of 80% Acetonitrile, 20% Water, 0.1% Formic Acid. Sample peptides were re-suspended in a buffer of 4% *v*/*v* Acetonitrile and 0.1% Formic Acid and injected in duplicate. An Eksigent system comprising of a nanoLC 400 autosampler along with a 425-pump module were used with a YMC-Triart C18 trap column and a YMC-Triart C18 analytical column. Mass spectra were obtained in SWATH mode and utilising the 100 variable window methods with optimised collision energy equations. 

The resultant spectral data files were converted using wiffconverter (Sciex, Warrington, UK)” and searched against our consensus spectral library using OpenSwath (version 2.0.0). Peptide matches were statistically scored using pyProphet (version 0.18.3) within the TransProteomic Pipeline (TPP) and then aligned using the TRIC tool from the OpenSWATH pipeline.

Downstream statistical analysis was performed using the Bioconductor (release 3.5) packages SWATH2Stats and MSstats within the R language (version 3.4.1). Gene ontology analysis was conducted using the webserver, WebGestalt [22]. Potential contaminants and decoy sequences were excluded prior to all analyses. To provide further insights into the nature of the spectral libraries, we produced sub-libraries based on specific sample types, thus allowing for comparison between libraries.

## 3. Results

### 3.1. Descriptive Characteristics of the Study Population

The demographic and clinical characteristics of the women whose cervico-vaginal fluid samples were used for the spectral library generation are summarised in Table 2. Cervico-vaginal samples from 19 women, comprising 9 EC cases, 3 AH’s and 7 controls, were used to generate the library. Five of the cases were endometrioid in histology while the remainder were non-endometrioid and included serous, clear cell, carcinosarcoma and mixed histology. All participants in this cohort were post-menopausal, with median age and BMI of 60 years (interquartile range, IQR 54–73 years) and 29 kg/m^2^ (IQR 27–38 kg/m^2^) respectively.

### 3.2. The Spectral Libraries

**Figure 3:**
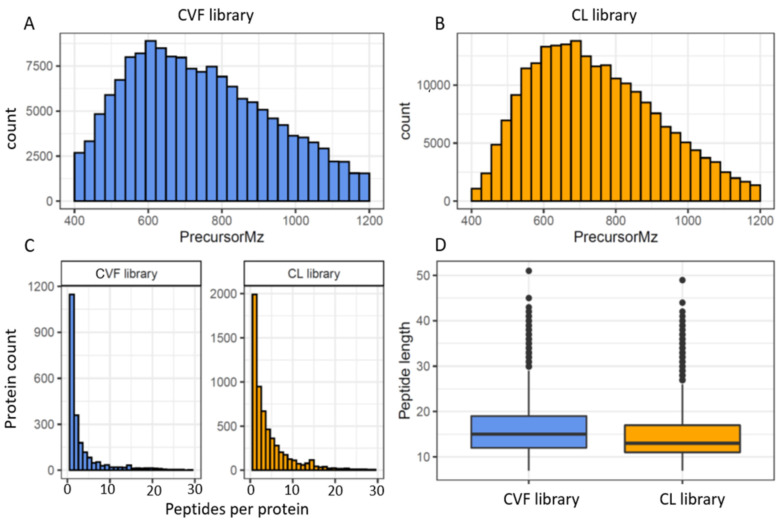
Overview of the cervico-vaginal fluid (CVF) and the EC cell-line (CL) based spectral library properties (**A**) histogram of the precursor ions *m/z* distribution of the CVF library (**B**) histogram of the precursor ions *m/z* distribution of the CL library (**C**) histogram of protein identifications based on the number of peptides per protein for both libraries (**D**) histogram of the peptide length distribution for both libraries.

The consensus cervico-vaginal fluid spectral library contains a total of 154,206 transitions and 23,886 precursors identifying 19,394 peptides and 2425 proteins. The spectral library based on the EC cell lines, on the other hand, comprised 221,844 transitions, 30,876 precursors, 29,384 peptide sequences and 6003 proteins. The coverage and characteristics of these assays are summarised in Table 3 and Figure 3.

As expected, the cervico-vaginal fluid library precursor ion *m/z* ranged between 400 *m/z* and 1200 *m/z* (median 726.3 *m/z*), in keeping with the MS mass range settings. A mass window of 400 to 1200 Da (*m/z*) is commonly employed in SWATH/DIA-MS analysis in order to obtain a full coverage of peptides. The product ion *m/z*, on the other hand, ranged between 350 *m/z* and 1998 *m/z* (median of 915 *m/z*). About 56% (13,401) of the precursor ions were doubly charged and the remainder (10 485, 43.9%) had a charge of 3. The fragment ions were mostly y type (132, 043, 85.6%) and singly charged (132, 038, 85.6%). The median peptide length of the peptide sequences was 15 amino acids (IQR 12, 19), and is consistent with the properties of full tryptic peptides. The average number of peptides per protein was 8 (median 2). Protein identification was based on the presence of a proteotypic peptide in 76% (1837) of proteins. The EC cell line-based assay had many properties similar to the cervico-cervico-vaginal fluid spectral library. The median peptide length of the cell line library was 13 amino acids (range 7–49, IQR 6) with 75% of peptide sequences having a length between 7 and 17. The median number of peptides per protein was 3. Approximately 75% of the cell lined based precursor ion *m/z* and product ion *m/z* ranged between 400–890 *m/z* and 350–1235 *m/z* respectively. Most precursor ions also had a charge of 2 while majority of the fragment ions were singly charged.

**Figure 4:**
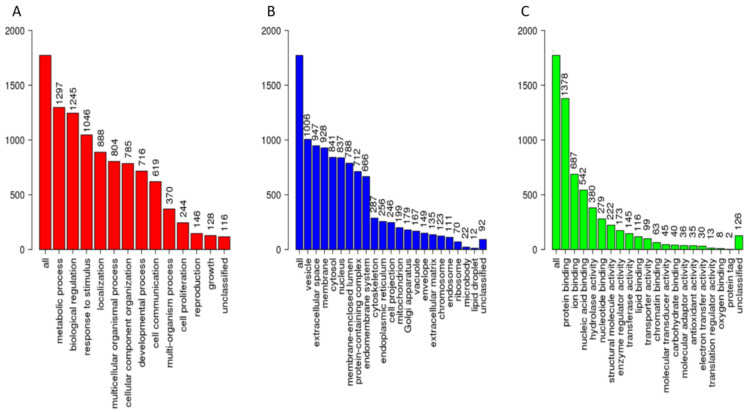
Gene ontological analyses of the cervico-vaginal fluid library proteotypic proteins mapped using the WebGestalt [22] Webserver, Available online: www.webgestalt.org (accessed on 10 May 2021). The biological processes (**A**), cellular processes (**B**) and molecular function categories (**C**) are as shown. The height of each bar represents the number of mapped IDs per category.

The cervico-vaginal fluid library includes a broad spectrum of regulatory, cytoskeletal and immune proteins. The gene ontology classification of the constituent proteins based on protein class, biological function, and molecular properties are summarised in Figure 4. Most of the library proteins had binding, structural and regulatory properties mediating cellular and metabolic functions. Approximately 50% of proteins were extra-cellular in origin.

**Figure 5:**
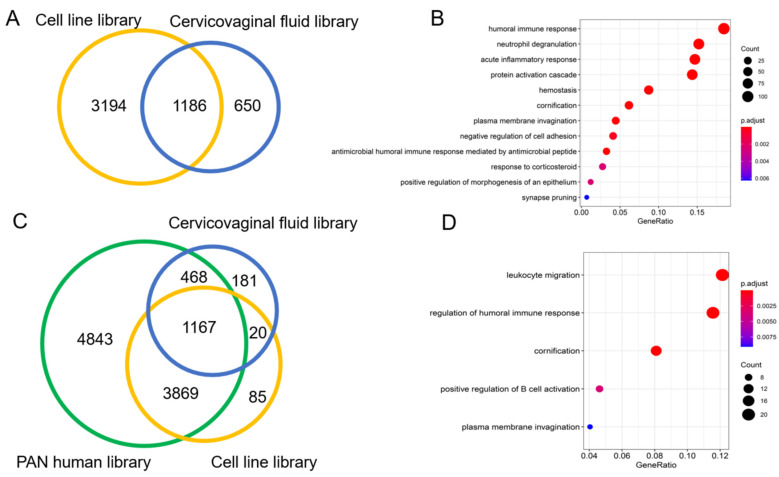
(**A**): Venn diagram showing overlap between the cervico-vaginal fluid and the cell line spectral libraries. (**B**). Gene ontological analysis (biological function) of the 650 proteins unique to the cervico-vaginal fluid library. (**C**). Venn diagram showing overlap between the cervico-vaginal fluid, EC cell line and pan Human libraries. (**D**). Gene ontological analysis (biological function) of the proteins unique to the cervico-vaginal fluid library. All analyses were based on proteotypic proteins.

The cervico-vaginal fluid and cell line spectral libraries showed a 20% commonality (1186) in identified proteins. About 11.2% (650) proteins were found to be unique to the cervico-vaginal fluid library (Figure 5A). These proteins were noted to be extracellular in origin and with immune-related and acute inflammatory properties (Figure 5B). A Venn diagram showing the overlap between both spectral libraries and the Pan Human library is shown in Figure 5B. Both spectral libraries captured 53% (5503) of the pan Human library proteins. The cervico-vaginal fluid spectral library contained 202 distinct proteins not present in the pan Human library while the cell line-based library contained 105 distinct proteins (Figure 5C). Gene Ontology analysis of the cervico-vaginal proteome unique proteins is shown in Figure 5D and reveals leucocyte migration, regulation of humoral immune response and cornification as their main biological functions. Gene names of proteins unique to the cervico-vaginal fluid library compared with the pan Human library are summarized in Table S1.

**Figure 6:**
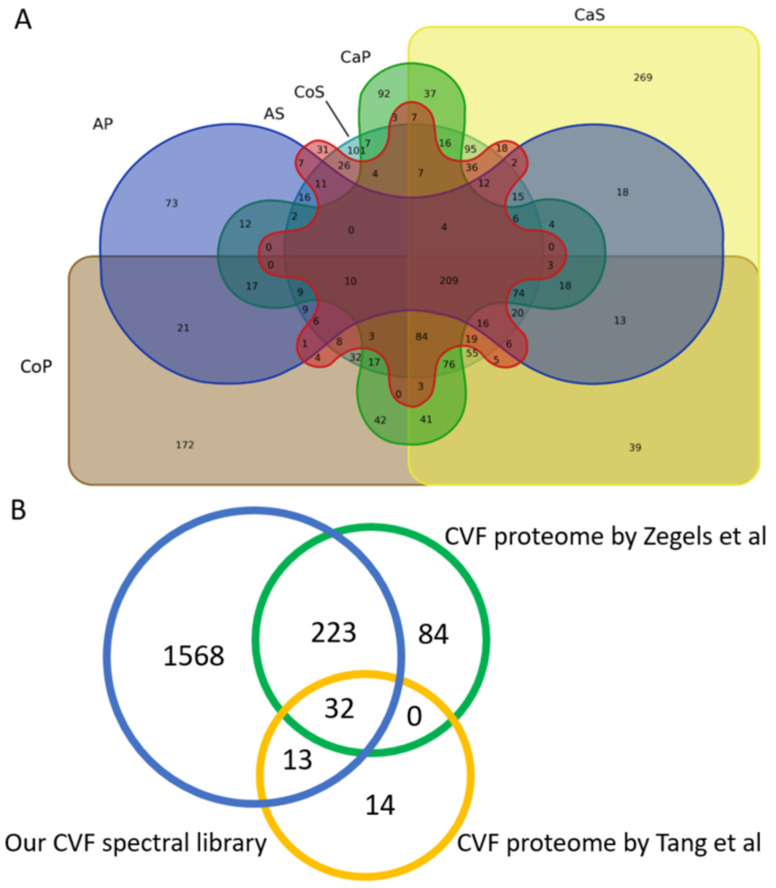
(**A**) Overview of the overlap of the various components that make up the consensus cervico-vaginal fluid library. AP: Atypical hyperplasia cell pellets, AS: Atypical hyperplasia supernatant, CaS: Cancer supernatant, CaP: Cancer pellets, CoS: Control Supernatant, CoP: Control pellets. All analyses were conducted based on proteins identified by proteotypic peptides. (**B**) Overview of the overlap between the cervico-vaginal fluid consensus spectral library and the cervico-vaginal fluid proteome described by Zegels et al. and Tang et al. respectively.

The cervico-vaginal fluid consensus spectral library comprised of proteins originating from the supernatant and cell pellet components of the cervico-vaginal fluid obtained from symptomatic controls, women with AH and EC. A Venn diagram showing the contribution of these various components in the generated library is shown in Figure 6. We found a total of 269 proteins to be unique to the cancer supernatant samples and 92 proteins to the cancer pellet samples. 37 unique proteins overlapped between the cancer pellets and supernatants. 31 proteins were unique to the AH supernatant samples and 73 proteins to the AH pellet samples. 

Next, we sought to compare the coverage of our cervico-vaginal fluid spectral library with the cervico-vaginal fluid proteome reported in previous studies. We did not identify any studies describing the cervico-vaginal fluid proteome of post-menopausal women with or at risk of endometrial cancer. A comparative overview of the various studies conducted on the cervico-vaginal fluid proteome in non-pregnant females is summarised in Table 4. A Venn diagram of the overlap between our spectral library and two other cervico-vaginal fluid proteome studies in non-pregnant women is shown is Figure 6. Our cervico-vaginal fluid library captured approximately 75% of the proteins described by Zegels et al. [23] and 76% of the 59 unique cell-free cervico-vaginal fluid proteins described by Tang et al. [24]. We were unable to assess the coverage of the proteome described by Shaw et al. due to lack of relevant data. Previously reported EC biomarker candidates captured in the library are summarised in Table S1, while other tumour-related proteins captured in the library are summarised in Table S2.

### 3.3. Spectral Library Validation

The library validation was carried out using cervico-vaginal fluid samples obtained from an independent cohort of women and comprised of five women at-risk of EC (technical validation) and ten women, including five with histologically confirmed EC and five controls (clinical validation). The technical validation cohort had a median age and BMI of 55 years (IQR 51–56 years) and 34 kg/m^2^ (19–37 kg/m^2^) respectively while the clinical validation cohort had a median age and BMI of 58 years (IQR 52–62 years), and 32 kg/m^2^ (27–36 kg/m^2^) respectively. The demographic and clinical characteristics of the validation cohorts are summarised in Table 5.

#### 3.3.1. Technical Validation and Applicability for SWATH-MS Data Analysis

**Figure 7:**
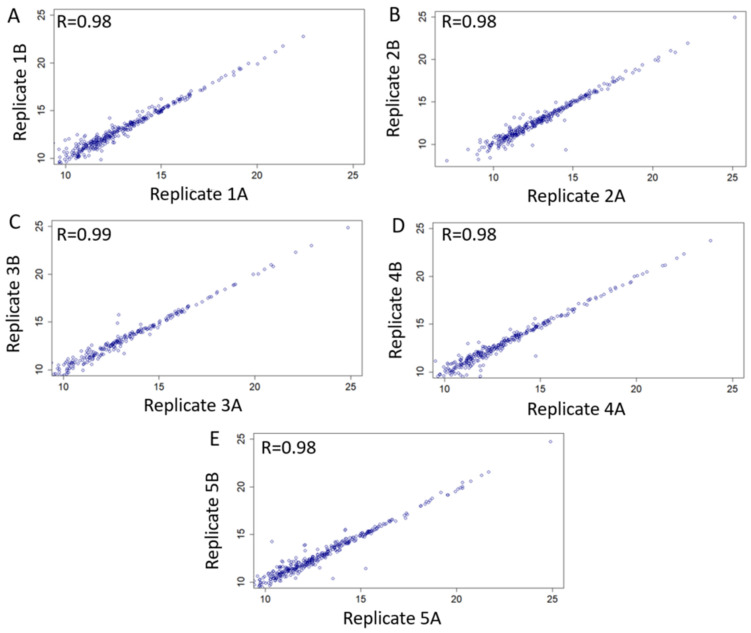
Technical validation and applicability of consensus cervico-vaginal fluid spectral library based on five samples obtained from an independent cohort of post-menopausal women at risk of endometrial cancer. Scatter plots of intensity correlation between replicates of all five validationsamples (**A**–**E**) using Pearson’s correlation analyses are as shown.

We searched our library using five cervico-vaginal fluid samples obtained from an independent cohort of post-menopausal women at-risk of EC (Table 5) and processed using standard SWATH-MS protocol, to assess the performance of our library. Each sample was run in duplicate. We evaluated the extent to which biological replicates were reproducible based on our spectral library using correlation analyses of normalised replicate values as shown in Figure 7. There was a very strong evidence of intensity correlation between sample replicates with Pearson’s correlation coefficient values of ≥0.98. 

**Figure 8:**
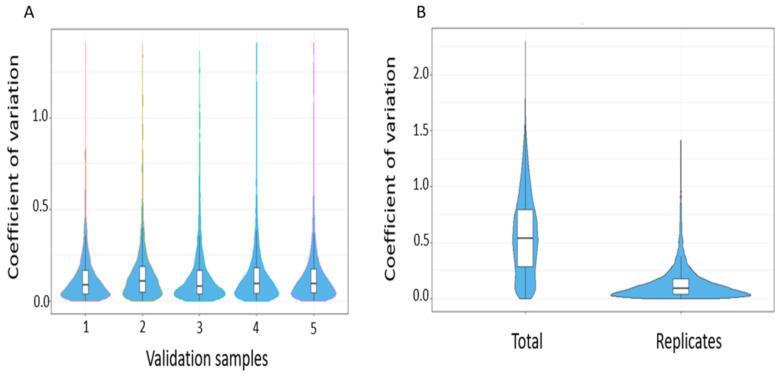
(**A**) Intra-assay coefficient of variation across validation samples (**B**) Summary coefficient of variation comparing total versus within replicates.

The reproducibility of peptide identifications was subsequently summarised using coefficient of variations of transition intensities at a 1% peptide FDR. The median intra-assay coefficient of variation for all replicates were <10 and is in keeping with expected technical variability (Figure 8). 

High rates of missingness are a major concern in proteomic studies [26,27]. We therefore sought to assess the rate of missing values between technical replicates of the same samples (Table 6). On average, the percentage of missing values in technical replicates was <10% and compares favorably to DDA proteomics where rates of missingness of at least 20% have been reported [28]. The overall rate of missingness per sample ranged between 12.9% and 21.8%, and is consistent with other label free mass spectrometry methods with rates of 10–50% [26,29].

#### 3.3.2. Real World Application of Spectral Library for Biomarker Discovery

**Figure 9:**
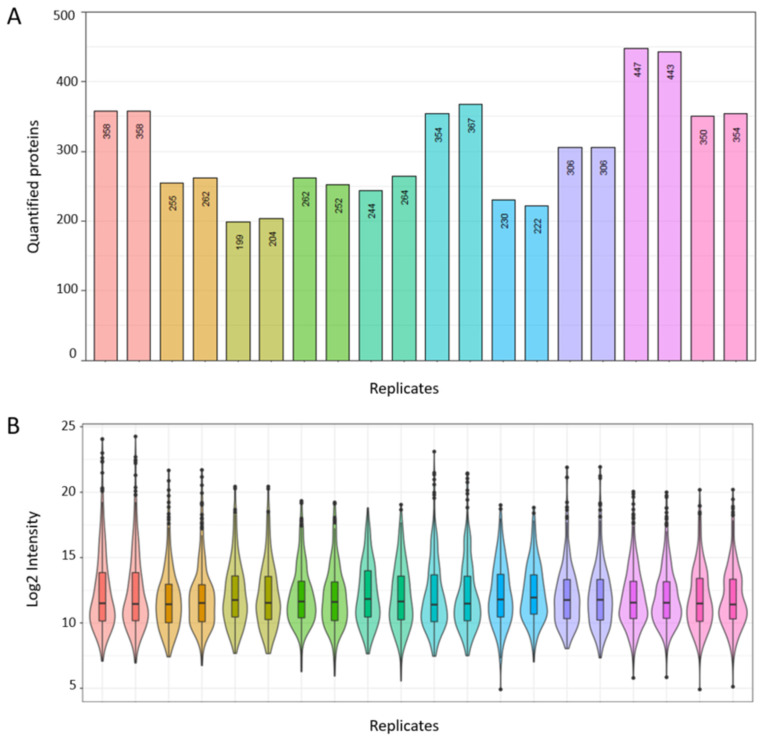
Real world application of spectral library for biomarker discovery based on cervico-vaginal fluid samples from five cancers and five controls. (**A**) Number of quantified proteins per study sample replicate. (**B**) Log 2 Intensity plot of corresponding sample replicates.

Next, we sought to provide evidence for the potential utility of our consensus spectral library for EC biomarker discovery in the real-world setting. Cervico-vaginal fluid samples from five cancer cases and five controls were processed and analysed in duplicate using our previously described standardised SWATH-MS protocol. The demographic and clinical characteristics of this validation cohort are summarised in Table 5. We then searched the obtained dataset against our consensus spectral library. Globally, a total of 680 proteins based on proteotypic peptides were identified in our validation dataset. The numbers of proteins quantified per sample replicate is summarised in Figure 9A, with identified protein counts ranging from 199 to 447. A Log2 intensity plot of sample replicates is shown in Figure 9B, with intensity correlation coefficients ranging from 0.95 to 0.99 (Figure S1). The coefficient of variations of transition intensities between replicates is summarised in Figure S2 with median CVs ranging from 7.8 to 10.9, in keeping with expected technical variations. 

**Figure 10:**
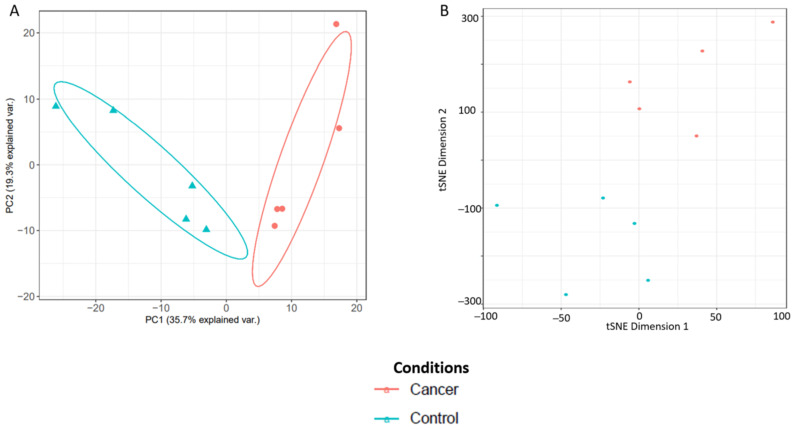
Analysis of sample separation comparing five EC cases and five controls using principal component analysis (PCA) (**A**) and t-distributed stochastic neighbour embedding (t-SNE) (**B**) plots based on differentially expressed cervico-vaginal fluid proteins.

Principal component analysis (PCA) and t-distributed stochastic neighbour embedding (t-SNE) plots were then used to assess degree of separation between groups and shown in Figure 10. The list and gene ontology analyses of the cervico-vaginal fluid proteins from the validation cohort used in generating the PCA and t-SNE plots are summarized in Table S2 and Figure S3 respectively. The differentially expressed proteins between the 5 cancer and 5 control samples are summarized in Table S3. Next, we selected five EC biomarker candidates previously reported in the literature and assessed how well the spectral library could reproducibly quantify them. The selected biomarkers were Human Epididymis Protein 4 (HE4/WFDC2), Cancer Antigen 15-3 (MUC 1), Matrix metalloproteinase 9, Fatty Acid Binding Protein-5 (FABP5) and Alpha-1B-Glycoprotein. All five biomarker candidates were reproducibly quantified with replicate intensity correlation coefficients of 0.99 respectively. The coefficient of variation across replicates of these biomarker candidates ranged from 0.62 to 4.88, again, in keeping with expected technical variations. (Table 7).

**Figure 11:**
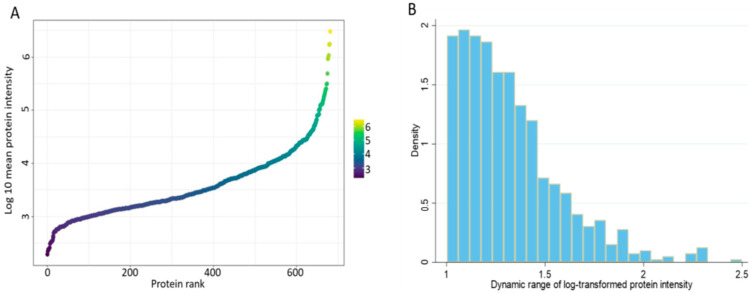
(**A**) Dynamic range of the cervico-vaginal fluid proteome based on the rank by abundance of the log10 intensity of all identified proteins in the validation cohort. (**B**) Dynamic ranges of individual proteins detected in the validation cohort based on the ratio of the maximum and minimum log10 protein intensities.

Finally, we sought to describe the dynamic range of the cervico-vaginal fluid proteome by ranking all identified proteins (log 10 mean protein intensity) in the validation cohort according to their abundance (Figure 11A). The cervico-vaginal fluid proteome spans about four orders of magnitude, in keeping with the proximal nature of the cervico-vaginal fluid and in contrast to the dynamic range of cancer-related proteins in blood, which is wider (plasma dynamic range ≥ 10 orders of magnitude) [30]. Next, we computed the dynamic ranges of individual proteins detected in the validation cohort based on the ratio of the maximum and minimum log10 protein intensities (Figure 11B). The identified proteins have dynamic range values ranging from 1.002 to 2.50 (SD 0.26) orders of magnitude.

## 4. Discussion

Proteomic analysis of human biological fluids has the potential to facilitate the discovery of clinically relevant biomarkers [31]. Cervico-vaginal fluid is a complex mixture of vaginal, endocervical, endometrial and fallopian tube secretions. Other components of cervico-vaginal fluid include exfoliated cells, commensal bacterial products, plasma transudate through the vaginal wall and secretions from vaginal immune cells [32]. Cervico-vaginal fluid exhibits antimicrobial properties and plays an important role in the immunity of the lower female genital tract. It is also responsible for maintaining homeostasis of the vaginal micro-environment [33,34]. Multiple studies have explored the potential of cervico-vaginal fluid to aid the detection of pregnancy associated pathologies such as pre-term birth [35,36,37,38], inflammatory/infective conditions of the lower genital tract [39,40] and cervical pre-cancer [41,42,43]. The anatomical continuity between the uterine cavity and the lower genital tract provides an opportunity for the sampling of uterine derived proteins and malignant cells using minimally invasive methods [13,44]. A recent study from our group showed proof-of-principle that cervico-vaginal fluid from women with EC contains malignant cells and is a viable source of EC biomarkers [45]. However, the potential utility of cervico-vaginal fluid as a source of protein biomarkers for the detection of EC, has been under-studied.

To our knowledge, our cervico-vaginal fluid consensus spectral library is the first and most comprehensive catalogue of proteins in cervico-vaginal fluid of women with or at-risk of EC. We identified 2425 proteins in pooled cervico-vaginal samples covering the full spectrum of EC, from symptomatic post-menopausal women, with no evidence of cancer, to those with AH and early and late-stage EC. Both endometrioid and non-endometrioid tumours were reflected in the library. The consensus library was built based on cervico-vaginal fluid supernatant proteins and proteins originating from exfoliated cells/endometrial malignant cells shed through the cervix into the lower genital tract [45]. The subsequent validation of the library using cervico-vaginal fluid samples from an independent cohort of women showed the library to be fit-for-purpose. Importantly, the library was robust in reproducibly quantifying selected EC biomarker candidates. Our library thus has the potential to enable the identification of protein biomarkers for the screening and early detection of EC.

Our cervico-vaginal fluid consensus spectral library makes an important contribution, in that over 200 proteins were found to be unique to this library, when compared with the Pan Human library. We did not find any previous studies describing the cervico-vaginal proteome of post-menopausal women or those with EC. A comparison with two previously reported comprehensive studies on the cervico-vaginal fluid proteome showed our cervico-vaginal fluid library capturing approximately 75% of the proteins described by Zegels et al. [23] and 76% of the 59 unique cell-free cervico-vaginal fluid proteins described by Tang et al. [24]. In addition, a large number of proteins were noted to be unique to our spectral library when compared with both previously reported proteomes. The Zegels study was relatively small, with just seven women, aged between 37 and 45 years, all of whom were either in the first- or second half of the menstrual cycle and with cervical pre-cancer (low-grade squamous intra-epithelial cervical lesions) [23]. The proteins unique to Zegels cervico-vaginal fluid proteome may therefore be related to the pre-menopausal status of the study participants or the presence of cervical pre-cancer. Tang et al., on the other hand, collected samples from 29 women, aged 24–48 years, seven of whom had evidence of candida [24]. Shaw and colleagues, using five clinical samples, characterised the cervico-vaginal proteome of young, healthy pre-menopausal women and identified 685 proteins, 30% of which were extracellular or membrane in origin [25]. A few other studies have explored the cervico-vaginal fluid proteome, but these have largely been in the context of menstrual cycle changes, pregnancy or cervical pre-cancer [23,25,46,47,48].

Our cervico-vaginal fluid consensus spectral library captures a broad range of proteins, many of which have previously been reported as EC biomarker candidates (Table S4). Of particular interest are the heat shock proteins/chaperones (hsp10, hsp60, hsp71, hsp75), a group of proteins that regulate protein folding and cell signalling and have been implicated in the proliferation and differentiation of tumour cells [49]. There is good evidence to suggest that these proteins are over-expressed in EC tissue specimens, although their utility as EC biomarkers may be limited by their non-specificity [50,51,52]. Other tissue-based EC biomarker candidates previously reported in the literature and captured in the library include calcium binding proteins (calgranulin (S-100A8/9), fatty acid binding proteins (putative fatty acid-binding protein 5-like protein 3), glycolytic enzymes (pyruvate kinase, Phosphoglycerate mutase 1, Alpha-enolase) and enzyme inhibitors (alpha1 antitrypsin). Calcium and fatty-acid binding proteins are involved in cell differentiation, proliferation, migration and apoptosis, while the over-expression of glycolytic enzymes in EC tissue specimens is linked to their role in generating ATP during cell proliferation [53,54].

Several plasma-based EC biomarker candidates were also captured in this library and include adipokines (e.g., adiponectin), plasma lipoproteins (apolipoproteins, plasma amyloid A), matrix metalloproteinases (MMP-9) and cancer-associated antigens (MUC 1, MUC 16 etc). Other plasma proteins include human epididymis protein-4 (HE-4), Alpha-1B-Glycoprotein, albumin, transferrin and immunoglobulins. The presence of these proteins in the cervico-vaginal fluid may relate to the transudation of plasma from the capillaries in the vaginal epithelium. A number of tumour related proteins were also noted (Table S5). As expected, proteins with antimicrobial functions were well represented including neutrophil defensins, cysteine-rich secretory protein 3, dermcidin, cathelicidin antimicrobial peptide, amongst others, as well as inflammatory markers such as C-reactive protein. 

This study has several strengths. Our spectral library reflects the full spectrum of EC and thus has the potential to identify and quantity both early (AH/stage 1A EC) and late-stage EC protein biomarkers. Early detection of EC is crucial for improved outcomes [13,55,56]. A recent study exploring the unmet research needs in EC found a detection tool that can triage symptomatic women for specialist review to be the 2nd most important research priority according to patients, carers and clinicians [57]. A similar study using James Lind Alliance methodology ranked “What simple, non-invasive, painless, cost-effective, and convenient tests can be used to detect cancer early?” first in the top ten research priorities for cancer early detection [58]. Whilst a detection tool based on SWATH-MS analysis of cervico-vaginal fluid samples may not be easily translated into routine clinical practice, it can pave the way for the development of a clinically tractable assay based on ELISA or Lumipulse ^®^ technology, or even lateral flow test technology for point of care testing. This approach can lead to rapid discrimination of cases and controls, with substantial cost-saving implications for healthcare providers, whilst saving women from invasive tests that are anxiety-provoking and avoidable in over 90% of cases. 

The validity and reliability of cervico-vaginal fluid sampling methodology directly influences the quality of cervico-vaginal fluid -based assay libraries. Of the four commonly used cervico-vaginal fluid collection methods, namely washings, swabs, wicks (including tampons) and diaphragm-like devices, cervico-vaginal washings, as used in this study, has shown superiority in terms of reproducibility and sample quality [59,60]. However, relevant markers are diluted in the washing buffer and sample optimization steps are often necessary during proteome assay development. Cervico-vaginal fluid collection using the Delphi device achieved excellent acceptability by patients [45,61] and ongoing work by our group is assessing it’s acceptability to practice nurses and general practitioners. Other strengths of this study include the choice of our control group which comprised women from a similar at-risk population as those with EC (i.e., symptomatic post-menopausal women) but who do not have EC. This constitutes the ideal control group for EC biomarker discovery studies, especially when compared to studies where healthy women, usually of younger age and without the symptom that needs investigating by the new test, are used as controls. Limitations of our study design include the relatively small number of representative samples of the various EC subtypes and stages. Also, we do not know how well the library will perform in identifying and quantifying relevant biomarkers in asymptomatic women or pre-menopausal women with hereditary predisposition to EC, as seen in Lynch syndrome. The differentially expressed proteins in the cervico-vaginal fluid of the five cancers and five controls are yet to be validated by immunoassays or targeted proteomics. Caution must be entertained when interpreting these results.

In order to enhance the utility of these datasets, we have provided relevant anonymised clinical and demographic information of the patient cohort whose samples were used in generating the library including age, tumour type, grade and stage. Our cell-line based library was built using Ishikawa and HEC-IA cell lines, representing pre- and post-menopausal endometrial cancer, respectively. Using the deposited data, bespoke cell-line spectral libraries that reflect the menopausal status of study participants may be recreated. Our datasets can be used to enrich and improve the comprehensiveness of other proteomic spectral libraries specifically tailored for the detection of gynaecological malignancies based on cervico-vaginal fluid sampling. The data set can also be further analysed using other state-of-the-art analytical software and may be integrated into larger EC related proteomic datasets and/or publically available protein libraries.

Our samples were processed using a TEAB lysis buffer and TCEP based digestion protocol. The generated library is compatible with other protocols, provided that lysis is complete, and reduction and alkylation of cysteine bonds are ensured. Although our library was generated based on 100 variable windows with selection of the six most intense fragment ions, spectral libraries based on a different window configuration and transition selection can be made using the deposited data. Furthermore, although the library was generated based on DDA data from an Orbitrap Fusion Lumos Tribrid mass spectrometer, it can still be used for data generated on different instruments. However, retention times will need to be re-aligned and collision energy settings optimised. Inclusion of iRT peptides [62,63] as commonly performed in DIA experiments gives opportunity in this respect to normalise data. Alternatively, conserved peptides can be used for retention time alignment [64]. Importantly, similarity of fragmentation spectra should be ensured by the optimization of collision energies.

## 5. Conclusions

In this study, we present the first comprehensive proteomic library with the potential to enable the identification and quantification of EC protein biomarkers in cervico-vaginal fluid. We identified 2425 proteins in pooled cervico-vaginal samples covering the full spectrum of EC. This is combined with a library of over 6000 proteins based on two EC cell lines. The libraries capture a broad range of proteins, including previously reported tissue and plasma-based EC biomarker candidates. Several proteins were noted to be unique to the consensus library when compared with both the Pan Human library and previously reported studies on the cervico-vaginal fluid proteome. Subsequent technical and clinical validation of the library using cervico-vaginal fluid samples from an independent cohort confirmed the library to be fit-for-purpose. Well-designed biomarker discovery studies with adequate sample sizes based on the use of SWATH-MS on cervico-vaginal fluid samples of women with and at-risk of EC are now needed to identify relevant biomarkers that can be translated into routine clinical practice.

## Figures and Tables

**Table 1:** Eligibility criteria for case and control selection.

Cases	Controls
Referred with postmenopausal bleeding	Referred with postmenopausal bleeding
Biopsy proven AH or EC	AH and EC excluded following routine diagnostic investigations, including TVS, biopsy and/or hysteroscopy
Able to give informed consent	Able to give informed consent
Sample taken prior to commencement of any treatment, including surgery, hormone therapy or chemotherapy	Sample taken prior to commencement of any treatment, including surgery or hormone therapy
	Can include those with benign pathologies such as benign polyp or atrophic vaginitis

**Table 2:** Demographic and clinical characteristics of cases and controls whose samples were used for library generation.

Serial Number	Age (Years)	BMI (kg/m^2^)	Diagnosis	Grade	Stage
**Library Generation Cohort**					
1	57	38	Atypical Hyperplasia	-	-
2	78	45	Atypical Hyperplasia	-	-
3	61	37	Atypical Hyperplasia	-	-
4	65	27	Endometrioid Endometrial cancer	1	1A
5	60	43	Endometrioid Endometrial cancer	1	1A
6	73	29	Endometrioid Endometrial cancer	2	1A
7	52	30	Endometrioid Endometrial cancer	2	1A
8	62	27	Clear Cell Endometrial cancer	3	1A
9	52	30	Serous Endometrial cancer	3	1A
10	74	27	Carcinosarcoma	3	1B
11	72	27	Mixed (Clear Cell/Endometrioid) cancer	3	2
12	84	28	Endometrioid Endometrial cancer	3	4B
13	54	29	Control (No endometrial pathology)	-	-
14	81	24	Control (No endometrial pathology)	-	-
15	56	24	Control (Atrophic vaginitis)	-	-
16	56	40	Control (No endometrial pathology)	-	-
17	52	44	Control (No endometrial pathology)	-	-
18	50	24	Control (No endometrial pathology)	-	-
19	56	24	Control (No endometrial pathology)	-	-

**Table 3:** Assay statistics for the consensus cervico-vaginal fluid and endometrial cancer cell-based spectral libraries at a protein FDR of 1%.

Assays	Proteotypic	Proteotypic and Shared
Consensus cervico-vaginal fluid spectral library		
Proteins	1836	2425
Peptides	18,247	19,394
Precursors	22,455	23,886
Transitions	144,060	154,206
Endometrial cancer cell-line based spectral library		
Proteins	5140	6003
Peptides	27,972	29,384
Precursors	29,509	30,876
Transitions	209,988	221,844

**Table 4:** Comparative overview of the various studies on the cervico-vaginal (CVF) proteome conducted in non-pregnant women.

Study Characteristics	Our CVF Spectral Library	CVF Proteome by Zegel et al. [23]	CVF Proteome by Tang et al. [24]	CVF Proteome by Shaw et al. [25]
**Sample size**	19	7	29	5
**Age range**	50–67 years	37–45 years	24–48 years	20–40 years
**Menopausal status**	Post-menopausal	Pre-menopausal	Pre-menopausal	Pre-menopausal
**Clinical diagnoses**	9 EC cases, 3 AH and 7 symptomatic women with no EC	All had cervical pre-cancer	Asymptomatic women, 7 had candida	Healthy female volunteers
**Sample collection**	Delphi screener (saline based wash)	Colposcopy (5% acetic acid wash)	Syringe (saline based wash)	Gauze (inserted in vagina for 1 h)
**Sample description**	Supernatant and pellets	Supernatant only	Supernatant only	Whole fluid
**Sample analysis**	Orbitrap Fusion Lumos Tribrid LC-MS	MALDI-TOF-TOF MS/MS	MALDI-TOF-TOF MS/MS	1D-SDS-PAGE, cation exchange, LS-MS/MS
**Protein identification**	X!Tandem	MASCOT	MASCOT	MASCOT & X!Tandem
**Spectral library protein count**	2425	339	147 (59 unique)	685

**Table 5:** Demographic and clinical characteristics of the technical and clinical validation cohorts.

Serial Number	Age (Years)	BMI (kg/m^2^)	Diagnosis	Grade	Stage
			Technical Validation Cohort		
20	50	19	Control (No endometrial pathology)		
21	52	37	Control (Benign endometrial polyp)		
22	55	34	Control (No endometrial pathology)		
23	56	19	Control (No endometrial pathology)		
24	56	37	Control (No endometrial pathology)		
			Clinical Validation Cohort		
25	58	36	Atypical Hyperplasia	-	-
26	67	30	Endometrioid endometrial cancer	1	1A
27	55	34	Endometrioid endometrial cancer	1	1A
28	50	54	Endometrioid endometrial cancer	1	1A
29	62	27	Clear cell	3	1A
30	60	58	Control (No endometrial pathology)	-	-
31	62	24	Control (Atrophic vaginitis)	-	-
32	58	30	Control (Benign endometrial polyp)	-	-
33	47	24	Control (No endometrial pathology)	-	-
34	52	33	Control (No endometrial pathology)	-	-

**Table 6:** Missing values between technical replicates of the same cervico-vaginal fluid samples.

Sample	Protein Count	Missing Values in Replicate 1	Missing Values in Replicate 2	Overall Rate
Sample 1	410	21(5.1%)	32(7.8%)	12.9%
Sample 2	389	25(6.4%)	28(7.2%)	13.6%
Sample 3	308	37(12.0%)	30(9.7%)	21.8%
Sample 4	378	29(7.7%)	33(8.7%)	16.4%
Sample 5	426	25(5.9%)	35(8.2%)	14.1%

**Table 7:** Reproducibility of selected biomarker candidates using the consensus cervico-vaginal fluid spectral library.

Biomarker Candidate	Gene Name	Intensity Correlation Coefficient	Coefficient of Variation
Human Epididymis Protein 4	HE4/WFDC2	0.99	3.29
Cancer Associated Antigen 15-3	MUC-1	0.99	4.88
Matrix Metalloproteinase 9	MMP9	0.99	2.01
Fatty Acid Binding Protein-5	FABP5	0.99	0.62
Alpha-1B-Glycoprotein	AIBG	0.99	1.36

## Data Availability

All datasets have been deposited in ProteomeXchange Consortium (http://proteomecentral.proteomexchange.org/ (accessed on 26 July 2021)) via the PRIDE partner repository with the dataset identifier PXD025925.

## Supplementary Materials

The following are available online at https://www.mdpi.com/article/10.3390/cancers13153804/s1. Figure S1: Intensity correlation coefficient values of the clinical validation cohort sample duplicates. Figure S2: The coefficient of variations of transition intensities between replicates for the clinical validation cohort. Figure S3: Gene ontology analyses of cervico-vaginal fluid proteins from the validation cohort used in generating the PCA and TSNE plots. Table S1: Gene names of proteins unique to cervico-vaginal fluid library compared to the pan-human library. Table S2: List of proteins detected in the cervico-vaginal fluid of the validation cohort and used for the PCA plots and TSNE plots in Figure 10. Table S3: Differentially expressed proteins between the five cancers and five controls used for library validation. Table S4: Previously reported EC biomarker candidates captured in the library. Table S5: Novel tumour related proteins captured in the library.
