# Supplementary material for: Comprehensive Library Generation for Identification and Quantification of Endometrial Cancer Protein Biomarkers in Cervico-Vaginal Fluid
Source: Cancers (Basel). 2021 Jul 28;13(15):3804. doi: 10.3390/cancers13153804 (PMC8345211; doi:10.3390/cancers13153804)
Supplement: Supplementary file 1 [file cancers-13-03804-s001.zip › cancers-1236363proof -supple.pdf]

Supplementary data

Figure S1: Intensity correlation coefficients of replicates of the 10 vaginal fluid samples used for clinical validation.

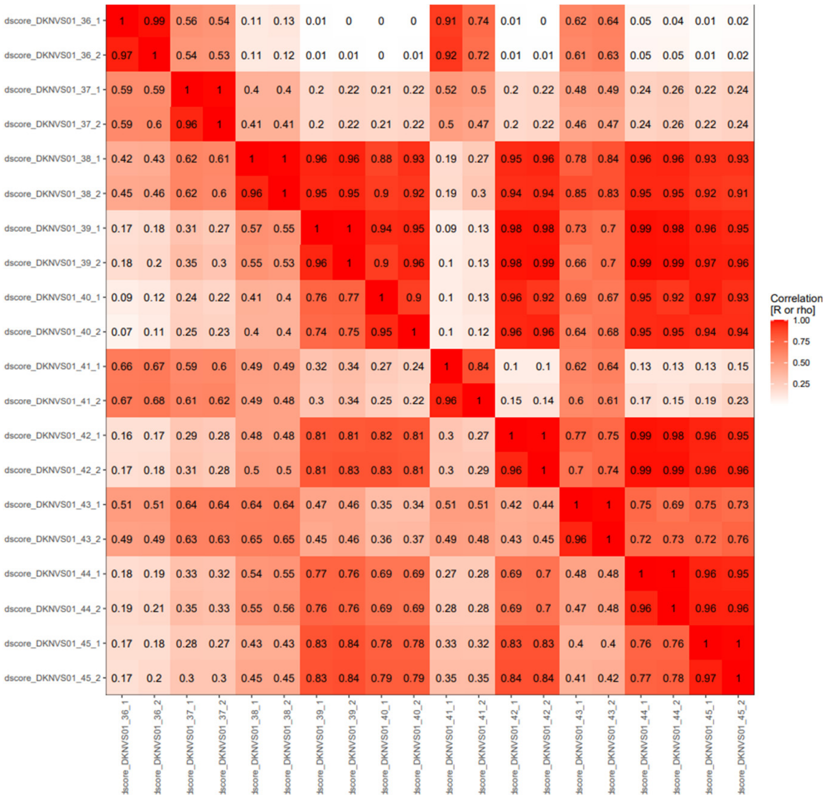

Figure S2: Coefficient of variations of transition intensities between replicates

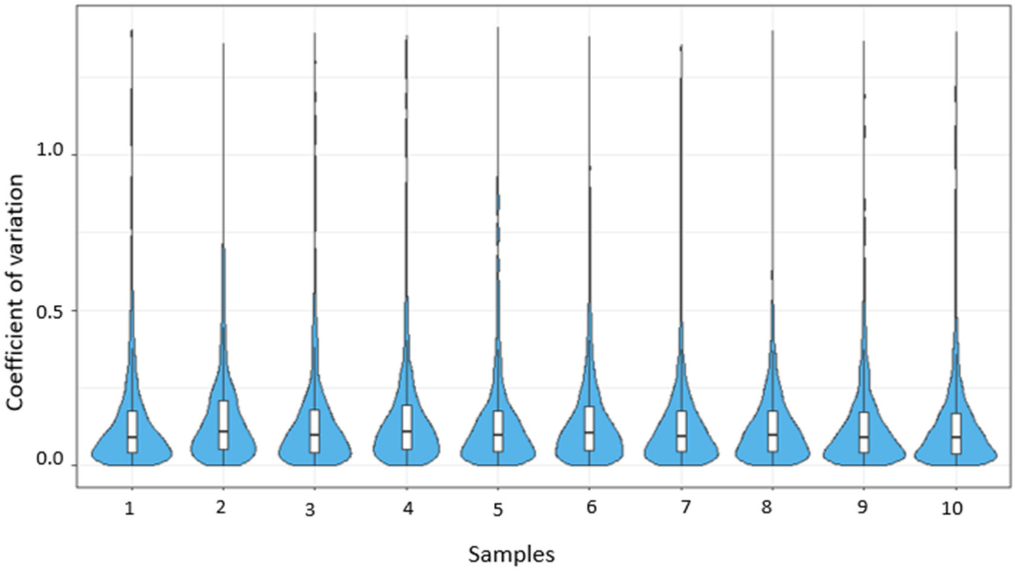

Figure S3: Gene ontology analyses of cervico-vaginal fluid proteins from the validation cohort used in generating the PCA and TSNE plots

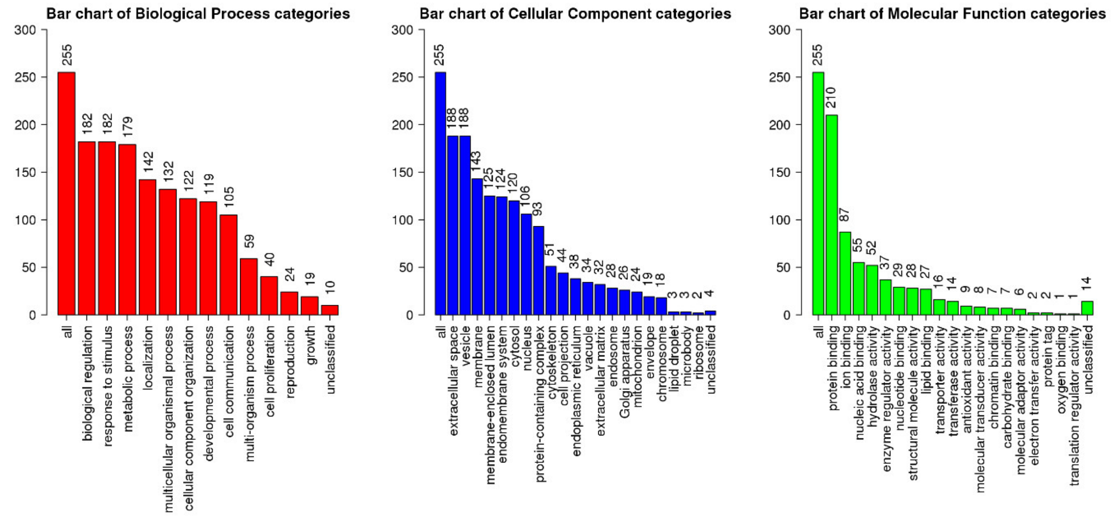

Table S1: Gene names of proteins unique to our cervico-vaginal fluid consensus spectral library compared to the pan-human library.

Gene names
ALPG
FAM43A
SOX11
TMPRSS11B
H2AC11
RNF121
MYL12B
CRNN
CDH23
TMPRSS11D
SERPINB13
DSG3
MMP7
ZSCAN22
SPINK5
ZNF716
ZNF7
ZNF250
ZNF30
ZBTB17
ZNF35
H2AC4
CAPNS2
VSIG10L
SPRR3
RPS17
ZBTB5
CYSRT1

TGM5
DLX4
RPTN
YIPF2
IGKV1-33
IGKV1-5
IGKV3-20
IGKV3-20
IGKV1-5
IGKV1-33
MUC21
GBP6
TUBA1B
SPRR1A
CLDN4
EMP1
ACTA2
TMPRSS6
NKX3-2
POU3F3
PAX2
ENDOU
ZBTB7C
PHOX2A
ACTC1
IGKV2D-28
KRT74
ZNF197
IGLV1-47
OBSCN
KLK13
KLK11
HOXB7
MAB21L4
RNASE7
ALPP
SKIL
TBX21
ACTG2
CPXCR1
MZF1
POTEE
IGKV3-15
LYPD2
SPRR1B
DUXA
IGHV3-23
IGHV3-30
HDX
TCP11L2
TMPRSS11A
UBB
ZSCAN5B
ZNF771
OSR2

CYP4F22
ZNF256
ALX4
ZNF438
TMPRSS11E
AIG1
SALL1
ZBTB40
PRR4
MYPOP
ZNF860
AMY1A
H2BC12
ALOX15B
H2AC18
ZFP92
IGKV4-1
PRSS27
DMKN
NME2P1
NPEPPSL1
MDK
DBX1
MMP25
CRCT1
ZNF652
ZNF550
PLA2G4D
TP73
ZNF500
ZNF407
BPIFB2
BHLHA9
SETBP1
IGKV3-20
TPRG1
CRYGS
DLX3
HOXC6
S100A7A
SPRR2A
H2BC18
SPRR2D
ZFPM1
GBP7
KLK5
MYC
IGKV1D-33
ASB15
MKX
IGHV3-13
TBX20
KRT72
N4BP2L1
IL36A

DNAH7
ZNF610
SP5
NR1D2
IGLC1
ZNF233
ZNF333
HIC2
THAP3
ANXA8
HAPLN3
IGLV1-51
IGLV1-40
IGLV1-47
FABP5P3
ZC3H3
IGHV3-33
IGHV1-69
IGHV1-46
IGHV1-69
IGHV3-11
IGHV3-30
ANXA2P2
HOXC4
H2AC6
IGLV3-1
MAPK1IP1L
EVX1
ZFP2
PRDM6
TSHZ3
TSHZ2
ZNF354C
TCF24
ZNF804B
ZNF350
KLF7
LY6D
RHOD
EBF3
ZNF777
KLK10
FAM25A
IGKV4-1
PAX7
STS
DLX6
RXRG
MAL
PROM2
GLI3
CA6
THRA
CREB3L2
CLDN1

ZFPM2
KRT12
MALL
FAM83A
IGKV1-17
IGLV3-1
MYOD1
FOXN1

Table S2: List of proteins detected in the cervico-vaginal fluid of the validation cohort

ID	Gene name	ID	Gene name	ID	Gene name	ID	Gene name
A8K2U0	A2ML1	P07384	CAN1	P35659	DEK	Q6E0U4	DMKN
O00151	PDLI1	P07437	TBB5	P35858	ALS	Q6FI13	H2A2A
O00160	MYO1F	P07476	INVO	P35908	K22E	Q6KB66	K2C80
O00231	PSD11	P07737	PROF1	P36222	CH3L1	Q6P4A8	PLBL1
O00299	CLIC1	P07814	SYEP	P36578	RL4	Q6UWP8	SBSN
O00391	QSOX1	P07858	CATB	P36952	SPB5	Q6UX06	OLFM4
O00505	IMA4	P07900	HS90A	P36955	PEDF	Q6UXB3	LYPD2
O00515	LAD1	P08107	HSP71	P37108	SRP14	Q6WKZ4	RFIP1
O14745	NHRF1	P08133	ANXA6	P37802	TAGL2	Q6XPR3	RPTN
O14791	APOL1	P08185	CBG	P37837	TALDO	Q6ZMR5	TM11A
O14818	PSA7	P08246	ELNE	P38159	RBMX	Q6ZN66	GBP6
O14880	MGST3	P08311	CATG	P38646	GRP75	Q6ZVX7	FBX50
O15144	ARPC2	P08519	APOA	P39023	RL3	Q7Z406	MYH14
O15231	ZN185	P08603	CFAH	P40121	CAPG	Q7Z4W1	DCXR
O15400	STX7	P08670	VIME	P40199	CEAM6	Q7Z794	K2C1B
O15511	ARPC5	P08697	A2AP	P40925	MDHC	Q86T26	TM11B
O43175	SERA	P08727	K1C19	P40926	MDHM	Q86U42	PABP2
O43240	KLK10	P08729	K2C7	P40939	ECHA	Q86V81	THOC4
O43707	ACTN4	P08758	ANXA5	P41218	MNDA	Q86X76	NIT1
O43747	AP1G1	P08779	K1C16	P43251	BTD	Q8IZP0	ABI1
P35579	MYH9	P08865	RSSA	P43490	NAMPT	Q8N122	RPTOR
O43760	SNG2	P08962	CD63	P43652	AFAM	Q8N1N4	K2C78
O43768	ENSA	P09211	GSTP1	P46776	RL27A	Q8NE71	ABCF1
O43852	CALU	P09237	MMP7	P46781	RS9	Q8NFJ5	RAI3
O43866	CD5L	P09382	LEG1	P46782	RS5	Q8TDL5	BPIB1
O60218	AK1BA	P09429	HMGB1	P46940	IQGA1	Q8TE68	ES8L1
O60235	TM11D	P09467	F16P1	P47756	CAPZB	Q8TEA8	DTD1
O60361	NDK8	P09525	ANXA4	P47914	RL29	Q8WUM4	PDC6I
O60437	PEPL	P09651	ROA1	P47929	LEG7	Q8WVM8	SCFD1
O60635	TSN1	P09758	TACD2	P48594	SPB4	Q8WVV4	POF1B
O60664	PLIN3	P09871	C1S	P48637	GSHB	Q92597	NDRG1
O60701	UGDH	P09960	LKHA4	P48643	TCPE	Q92817	EVPL
O60784	TOM1	P09972	ALDOC	P48668	K2C6C	Q92820	GGH
O60814	H2B1K	P0C0L4	CO4A	P48735	IDHP	Q92876	KLK6
O60888	CUTA	P0C0L5	CO4B	P49006	MRP	Q92882	OSTF1
O75083	WDR1	P0CG05	LAC2	P49189	AL9A1	Q96AE4	FUBP1
O75223	GGCT	P0CG47	UBB	P49327	FAS	Q96C19	EFHD2
O75342							

O75347	LX12B	P10153	RNAS2	P49368	TCPG	Q96DA0	ZG16B
O75363	TBCA	P10412	H14	P49411	EFTU	Q96FQ6	S10AG
O75367	BCAS1	P10451	OSTP	P49419	AL7A1	Q96FW1	OTUB1
O75368	H2AY	P10599	THIO	P49720	PSB3	Q96G03	PGM2
O75369	SH3L1	P10643	CO7	P49913	CAMP	Q96HC4	PDLI5
O75390	FLNB	P10696	PPBN	P50395	GDIB	Q96HE7	ERO1A
O75531	CISY	P10809	CH60	P50452	SPB8	Q96KP4	CNDP2
O75556	BAF	P10909	CLUS	P50914	RL14	Q96P63	SPB12
O75594	SG2A1	P11021	GRP78	P50990	TCPQ	Q96PD5	PGRP2
O75629	PGRP1	P11142	HSP7C	P50991	TCPD	Q96TA1	NIBL1
O75874	CREG1	P11215	ITAM	P50995	ANX11	Q99102	MUC4
O75882	IDHC	P11279	LAMP1	P51149	RAB7A	Q99460	PSMD1
O76027	ATRN	P11413	G6PD	P51659	DHB4	Q99536	VAT1
O94919	ANXA9	P11586	C1TC	P51884	LUM	Q99623	PHB2
O95171	ENDD1	P12035	K2C3	P52209	6PGD	Q99714	HCD2
O95274	SCEL	P12111	CO6A3	P52565	GDIR1	Q9BPY8	HOP
O95817	LYPD3	P12273	PIP	P52566	GDIR2	Q9BQE3	TBA1C
O95833	BAG3	P12429	ANXA3	P52597	HNRPF	Q9BQI0	AIF1L
P00338	CLIC3	P12724	ECP	P52790	HXK3	Q9BQR3	PRS27
P00390	LDHA	P12814	ACTN1	P52907	CAZA1	Q9BRA2	TXD17
P00441	GSHR	P12830	CADH1	P52943	CRIP2	Q9BRF8	CPPED
P00450	SODC	P12838	DEF4	P53004	BIEA	Q9BRP8	WIBG
P00491	CERU	P12956	XRCC6	P54108	CRIS3	Q9BW04	SARG
P00505	PNPH	P13010	XRCC5	P54652	HSP72	Q9C002	NMES1
P00558	AATM	P13473	LAMP2	P54727	RD23B	Q9GZP8	IMUP
P00734	PGK1	P13489	RINI	P55058	PLTP	Q9GZX5	ZN350
P00736	THRB	P13639	EF2	P55072	TERA	Q9H0U4	RAB1B
P00739	C1R	P13645	K1C10	P55145	MANF	Q9H161	ALX4
P00747	HPTR	P13646	K1C13	P55327	TPD52	Q9H1E1	RNAS7
P00748	PLMN	P13647	K2C5	P56134	ATPK	Q9H299	SH3L3
P00751	FA12	P13671	CO6	P59998	ARPC4	Q9H2G2	SLK
P00915	CFAB	P13796	PLSL	P60174	TPIS	Q9HC84	MUC5B
P00918	CAH1	P13797	PLST	P60468	SC61B	Q9HCY8	S10AE
P01008	CAH2	P13861	KAP2	P60660	MYL6	Q9HD89	RETN
P01011	ANT3	P13987	CD59	P60709	ACTB	Q9HDC9	APMAP
P01019	AACT	P14174	MIF	P60842	IF4A1	Q9NQ38	ISK5
P01024	ANGT	P14317	HCLS1	P60866	RS20	Q9NQC3	RTN4
P01031	CO3	P14618	KPYM	P60900	PSA6	Q9NRE2	TSH2
P01034	CO5	P14625	ENPL	P60903	S10AA	Q9NVZ3	NECP2
P01037	CYTC	P14780	MMP9	P60953	CDC42	Q9NYU2	UGGG1
P01040	CYTN	P14868	SYDC	P60981	DEST	Q9NZD2	GLTP
P01042	CYTA	P14923	PLAK	P61019	RAB2A	Q9NZT1	CALL5
P01106	KNG1	P15104	GLNA	P61026	RAB10	Q9P1F3	ABRAL
P01266	MYC	P15311	EZRI	P61106	RAB14	Q9UBC9	SPRR3
P01591	THYG	P15428	PGDH	P61158	ARP3	Q9UBG3	CRNN
P01596	IGJ	P15924	DESP	P61160	ARP2	Q9UGL9	CRCT1
P01598	KV104	P15941	MUC1	P61163	ACTZ	Q9UGM3	DMBT1
P01603	KV106	P16070	CD44	P61604	CH10	Q9UHA7	IL36A

P01609	KV111	P16152	CBR1	P61626	LYSC	Q9UIV8	SPB13
P01610	KV117	P16401	H15	P61769	B2MG	Q9UJ70	NAGK
P01612	KV118	P16402	H13	P61916	NPC2	Q9UJU6	DBNL
P01617	KV120	P16403	H12	P61978	HNRPK	Q9UJY1	HSPB8
P01620	KV204	P16949	STMN1	P61981	1433G	Q9UK76	HN1
P01621	KV302	P16989	YBOX3	P62081	RS7	Q9UKR3	KLK13
P01622	KV303	P17096	HMGA1	P62158	CALM	Q9UL52	TM11E
P01625	KV304	P17174	AATC	P62258	1433E	Q9ULC6	PADI1
P01765	KV402	P17213	BPI	P62263	RS14	Q9ULD9	ZN608
P01770	HV304	P17931	LEG3	P62266	RS23	Q9UM07	PADI4
P01781	HV309	P17936	IBP3	P62318	SMD3	Q9UN36	NDRG2
P01833	HV320	P17987	TCPA	P62328	TYB4	Q9UQ35	SRRM2
P02042	PIGR	P18054	LOX12	P62424	RL7A	Q9Y2P7	ZN256
P02511	HBD	P18065	IBP2	P62753	RS6	Q9Y2V2	CHSP1
P02533	CRYAB	P18124	RL7	P62805	H4	Q9Y3Q3	TMED3
P02538	K1C14	P18135	KV312	P62826	RAN	Q9Y446	PKP3
P02545	K2C6A	P18206	VINC	P62847	RS24	Q9Y490	TLN1
P02649	LMNA	P18510	IL1RA	P62899	RL31	Q9Y4K1	AIM1
P02654	APOE	P18669	PGAM1	P62917	RL8	Q9Y5K6	CD2AP
P02655	APOC1	P19012	K1C15	P62937	PPIA	Q9Y5Z4	HEBP2
P02656	APOC2	P19013	K2C4	P62942	FKB1A	Q9Y6R7	FCGBP
P02730	APOC3	P19367	HXK1	P62995	TRA2B	Q5T0Z8	CF132
P02741	B3AT	P19823	ITIH2	P63104	1433Z		
P02743	CRP	P19827	ITIH1	P63244	GBLP		
P02746	SAMP	P19957	ELAF	P63261	ACTG		
P02747	C1QB	P20061	TCO1	P63267	ACTH		
P02748	C1QC	P20160	CAP7	P63313	TYB10		
P02749	CO9	P20591	MX1	P67936	TPM4		
P02750	APOH	P20618	PSB1	P68036	UB2L3		
P02751	A2GL	P20700	LMNB1	P68104	EF1A1		
P02753	FINC	P20810	ICAL	P68363	TBA1B		
P02760	RET4	P20930	FILA	P68366	TBA4A		
P02763	AMBP	P20962	PTMS	P68871	HBB		
P02765	A1AG1	P21333	FLNA	P69905	HBA		
P02766	FETUA	P21926	CD9	P78371	TCPB		
P02774	TTHY	P22234	PUR6	P80188	NGAL		
P02788	VTDB	P22314	UBA1	P80511	S10AC		
P02790	TRFL	P22528	SPR1B	P80723	BASP1		
P03952	HEMO	P22626	ROA2	P83731	RL24		
P03973	KLKB1	P22735	TGM1	P84098	RL19		
P04003	SLPI	P22792	CPN2	P98160	PGBM		
P04004	C4BPA	P22894	MMP8	Q00325	MPCP		
P04040	VTNC	P23142	FBLN1	Q00610	CLH1		
P04075	CATA	P23246	SFPQ	Q00796	DHSO		
P04080	ALDOA	P23284	PPIB	Q00839	HNRPU		
P04083	CYTB	P23381	SYWC	Q01469	FABP5		
P04114	ANXA1	P23396	RS3	Q01518	CAP1		
P04179	APOB	P23526	SAHH	Q01844	EWS		

P04196	SODM	P23528	COF1	Q02413	DSG1		
P04207	HRG	P24158	PRTN3	Q02487	DSC2		
P04208	KV308	P25205	MCM3	Q02878	RL6		
P04217	LV106	P25311	ZA2G	Q02978	M2OM		
P04220	A1BG	P25705	ATPA	Q04695	K1C17		
P04259	MUCB	P25787	PSA2	Q04837	SSBP		
P04264	K2C6B	P25788	PSA3	Q04917	1433F		
P04275	K2C1	P25789	PSA4	Q05315	LEG10		
P04406	VWF	P25815	S100P	Q06033	ITIH3		
P04434	G3P	P26038	MOES	Q06323	PSME1		
P04632	KV310	P26373	RL13	Q06830	PRDX1		
P04792	CPNS1	P26583	HMGB2	Q07020	RL18		
P04839	HSPB1	P26641	EF1G	Q07654	TFF3		
P04899	CY24B	P27105	STOM	Q07812	BAX		
P04908	GNAI2	P27169	PON1	Q08188	TGM3		
P05089	H2A1B	P27348	1433T	Q08380	LG3BP		
P05090	ARGI1	P27482	CALL3	Q09666	AHNK		
P05107	APOD	P27797	CALR	Q12802	AKP13		
P05109	ITB2	P27816	MAP4	Q12841	FSTL1		
P05120	S10A8	P27824	CALX	Q13011	ECH1		
P05141	PAI2	P28066	PSA5	Q13126	MTAP		
P05155	ADT2	P28072	PSB6	Q13228	SBP1		
P05156	IC1	P28074	PSB5	Q13442	HAP28		
P05160	CFAI	P28838	AMPL	Q13510	ASAH1		
P05164	F13B	P29373	RABP2	Q13751	LAMB3		
P05187	PERM	P29401	TKT	Q13813	SPTN1		
P05386	PPB1	P29508	SPB3	Q13835	PKP1		
P05387	RLA1	P29622	KAIN	Q13867	BLMH		
P05543	RLA2	P29692	EF1D	Q14002	CEAM7		
P05546	THBG	P29966	MARCS	Q14019	COTL1		
P05556	HEP2	P30041	PRDX6	Q14103	HNRPD		
P05783	ITB1	P30043	BLVRB	Q14134	TRI29		
P05787	K1C18	P30044	PRDX5	Q14152	EIF3A		
P06311	K2C8	P30048	PRDX3	Q14210	LY6D		
P06396	KV311	P30050	RL12	Q14247	SRC8		
P06454	GELS	P30085	KCY	Q14508	WFDC2		
P06576	PTMA	P30086	PEBP1	Q14624	ITIH4		
P06681	ATPB	P30101	PDIA3	Q14697	GANAB		
P06702	CO2	P30153	2AAA	Q14764	MVP		
P06727	S10A9	P30740	ILEU	Q14974	IMB1		
P06731	APOA4	P31146	COR1A	Q14CN2	CLCA4		
P06733	CEAM5	P31151	S10A7	Q15084	PDIA6		
P06737	ENOA	P31930	QCR1	Q15149	PLEC		
P06744	PYGL	P31944	CASPE	Q15181	IPYR		
P06748	G6PI	P31946	1433B	Q15365	PCBP1		
P06753	NPM	P31947	1433S	Q15393	SF3B3		
P06889	TPM3	P31949	S10AB	Q15582	BGH3		
P07099	LV405	P31997	CEAM8	Q15843	NEDD8		

Biomarker
1/sp|A8K2U0|A2ML1_HUMAN
1/sp|O60235|TM11D_HUMAN
1/sp|O75223|GGCT_HUMAN
1/sp|P00450|CERU_HUMAN
1/sp|P00734|THRB_HUMAN
1/sp|P00751|CFAB_HUMAN
1/sp|P01008|ANT3_HUMAN
1/sp|P01011|AACT_HUMAN
1/sp|P01019|ANGT_HUMAN
1/sp|P01024|CO3_HUMAN
1/sp|P01031|CO5_HUMAN
1/sp|P01040|CYTA_HUMAN
1/sp|P01042|KNG1_HUMAN
1/sp|P02042|HBD_HUMAN
1/sp|P02533|K1C14_HUMAN

P07108	HYEP	P32119	PRDX2	Q15847	ADIRF		
P07195	ACBP	P32320	CDD	Q16186	ADRM1		
P07225	LDHB	P32926	DSG3	Q16610	ECM1		
P07237	PROS	P34932	HSP74	Q16658	FSCN1		
P07339	PDIA1	P35221	CTNA1	Q16698	DECR		
P07355	CATD	P35237	SPB6	Q16831	UPP1		
P07357	ANXA2	P35268	RL22	Q16851	UGPA		
P07358	CO8A	P35321	SPR1A	Q32MZ4	LRRF1		
P07360	CO8B	P35326	SPR2A	Q53FA7	QORX		
	CO8G	P35527	K1C9	Q5D862	FILA2		

Table S3: Differentially expressed proteins between the five cancers and five controls used for library validation.

1/sp|P02649|APOE_HUMAN
1/sp|P02654|APOC1_HUMAN
1/sp|P02743|SAMP_HUMAN
1/sp|P02746|C1QB_HUMAN
1/sp|P02747|C1QC_HUMAN
1/sp|P02748|CO9_HUMAN
1/sp|P02751|FINC_HUMAN
1/sp|P02753|RET4_HUMAN
1/sp|P02760|AMBP_HUMAN
1/sp|P02763|A1AG1_HUMAN
1/sp|P02766|TTHY_HUMAN
1/sp|P02774|VTDB_HUMAN
1/sp|P02790|HEMO_HUMAN
1/sp|P04003|C4BPA_HUMAN
1/sp|P04004|VTNC_HUMAN
1/sp|P04114|APOB_HUMAN
1/sp|P04196|HRG_HUMAN
1/sp|P04217|A1BG_HUMAN
1/sp|P05090|APOD_HUMAN
1/sp|P05109|S10A8_HUMAN
1/sp|P05156|CFAI_HUMAN
1/sp|P05543|THBG_HUMAN
1/sp|P05546|HEP2_HUMAN
1/sp|P06727|APOA4_HUMAN
1/sp|P06731|CEAM5_HUMAN
1/sp|P07225|PROS_HUMAN
1/sp|P07355|ANXA2_HUMAN
1/sp|P07360|CO8G_HUMAN
1/sp|P08185|CBG_HUMAN
1/sp|P08603|CFAH_HUMAN

1/sp|P08697|A2AP_HUMAN
1/sp|P10643|CO7_HUMAN
1/sp|P10909|CLUS_HUMAN
1/sp|P11142|HSP7C_HUMAN
1/sp|P13671|CO6_HUMAN
1/sp|P19823|ITIH2_HUMAN

1/sp|P19827|ITIH1_HUMAN
1/sp|P22735|TGM1_HUMAN
1/sp|P22792|CPN2_HUMAN
1/sp|P27169|PON1_HUMAN
1/sp|P29622|KAIN_HUMAN
1/sp|P30740|ILEU_HUMAN
1/sp|P31151|S10A7_HUMAN
1/sp|P32926|DSG3_HUMAN
1/sp|P43251|BTD_HUMAN
1/sp|P43652|AFAM_HUMAN
1/sp|P49913|CAMP_HUMAN
1/sp|P51884|LUM_HUMAN
1/sp|P55058|PLTP_HUMAN
1/sp|P62805|H4_HUMAN
1/sp|P68871|HBB_HUMAN
1/sp|P69905|HBA_HUMAN
1/sp|P80188|NGAL_HUMAN
1/sp|Q01469|FABP5_HUMAN
1/sp|Q08188|TGM3_HUMAN
1/sp|Q08380|LG3BP_HUMAN
1/sp|Q14134|TRI29_HUMAN
1/sp|Q14210|LY6D_HUMAN
1/sp|Q14624|ITIH4_HUMAN
1/sp|Q6P4A8|PLBL1_HUMAN
1/sp|Q8TEA8|DTD1_HUMAN
1/sp|Q92876|KLK6_HUMAN
1/sp|Q96PD5|PGRP2_HUMAN
1/sp|Q9UIV8|SPB13_HUMAN
1/sp|Q9UKR3|KLK13_HUMAN

Table S4: Previously reported endometrial cancer biomarker candidates captured in the cervico-vaginal fluid consensus spectral library

Protein	Uniprot ID
Adiponectin	Q15848
Alpha-1-acid glycoprotein 1	P02763
Alpha-1-antitrypsin	P01009
Alpha-1B-glycoprotein	P04217
Alpha-enolase	P06733
Annexin A11	P50995
Annexin A3	P12429

Annexin A4	P09525
Annexin A5	P08758
Annexin A8	P13928
Antithrombin-III	P01008
Apolipoprotein A-IV	P06727
Apolipoprotein C-III	P02656
Apolipoprotein L1	O14791
Calreticulin	P27797
Complement C2	P06681
Complement C5	P01031
Costars family protein ABRAC	Q9P1F3
Creatine kinase B-type	P12277
Creatine kinase U-type, mitochondrial	P12532
Epidermal growth factor receptor kinase substrate 8-like protein 2	Q9H6S3
Fatty acid synthase	P49327
Fatty acid-binding protein 5	Q01469
Fatty acid-binding protein, adipocyte	P15090
Fibrinogen beta chain	P02675
Heat shock 70 kDa protein 4	P34932
Heat shock cognate 71 kDa protein	P11142
Heat shock protein 10 kDa, mitochondrial	P61604
Heat shock protein 60 kDa, mitochondrial	P10809
Heat shock protein 75 kDa, mitochondrial	Q12931
Heat shock-related 70 kDa protein 2	P54652
Heterogeneous nuclear ribonucleoprotein K	P61978
Hsp90 co-chaperone Cdc37	Q16543
Insulin-like growth factor-binding protein 3	P17936
Leucine-rich alpha-2-glycoprotein	P02750
L-lactate dehydrogenase B chain	P07195
Matrix metalloproteinase-25	Q9NPA2
Matrix metalloproteinase-9	P14780
Metalloproteinase inhibitor 1	P01033
Mucin-1	P15941
Mucin-21	Q5SSG8
Mucin-4	Q99102
Mucin-5AC	P98088
Mucin-5B	Q9HC84
Phosphoglycerate mutase 1	P18669
Pro-cathepsin H	P09668
Prohibitin	P35232
Protein S100-A9	P06702
Putative fatty acid-binding protein 5-like protein 3	A8MUU1
Pyruvate kinase	P14618
Serotransferrin	P02787
Serum amyloid A-4 protein	P35542
Stress-induced-phosphoprotein 1	P31948
Superoxide dismutase (Cu-Zn)	P00441
Superoxide dismutase (Mn), mitochondrial	P04179
Transgelin-2	P37802
Transthyretin	P02766
WAP four-disulfide core domain protein 2	Q14508

Table S5: Tumour related proteins captured in the cervico-vaginal fluid consensus spectral library.

Protein	Uniprot ID
Apoptosis regulator BAX	Q07812
BH3-interacting domain death agonist	P55957
Breast carcinoma-amplified sequence 1	O75363
Cell division control protein 42 homolog	P60953

DNA replication licensing factor MCM5	P33992
DNA replication licensing factor MCM6	Q14566
DNA replication licensing factor MCM7	P33993
Hypermethylated in cancer 2 protein	Q96JB3
Hypoxia up-regulated protein 1	Q9Y4L1
Migration and invasion enhancer 1	Q9BRT3
Myc proto-oncogene protein	P01106
Programmed cell death 6-interacting protein	Q8WUM4
Programmed cell death protein 10	Q9BUL8
Programmed cell death protein 6	O75340
Prolactin-inducible protein	P12273
Proliferation-associated protein 2G4	Q9UQ80
Protein FAM3C	Q92520
Proto-oncogene c-Rel	Q04864
Ras-related protein Rab-5C	P51148
Translationally-controlled tumor protein	P13693
Tumor protein D52	P55327
Tumor protein D54	O43399
Tumor protein p73	O15350
